# “One-Size-Fits-All”? Optimizing Treatment Duration for Bacterial Infections

**DOI:** 10.1371/journal.pone.0029838

**Published:** 2012-01-11

**Authors:** Patricia Geli, Ramanan Laxminarayan, Michael Dunne, David L. Smith

**Affiliations:** 1 Center for Disease Dynamics, Economics and Policy, Washington, D.C., United States of America; 2 Princeton Environmental Institute, Princeton, New Jersey, United States of America; 3 Durata Therapeutics, Inc., Morristown, New Jersey, United States of America; 4 Department of Zoology and Emerging Pathogens Institute, University of Florida, Gainesville, Florida, United States of America; Dana-Farber Cancer Institute, United States of America

## Abstract

Historically, antibiotic treatment guidelines have aimed to maximize treatment efficacy and minimize toxicity, but have not considered the evolution of antibiotic resistance. Optimizing the duration and dosing of treatment to minimize the duration of symptomatic infection and selection pressure for resistance simultaneously has the potential to extend the useful therapeutic life of these valuable life-saving drugs without compromising the interests of individual patients.

Here, using mathematical models, we explore the theoretical basis for shorter durations of treatment courses, including a range of ecological dynamics of bacteria that cause infections or colonize hosts as commensals. We find that immunity is an important mediating factor in determining the need for long duration of treatment. When immunity to infection is expected, shorter durations that reduce the selection for resistance without interfering with successful clinical outcome are likely to be supported. Adjusting drug treatment strategies to account for the impact of the differences in the ecological niche occupied by commensal flora relative to invasive bacteria could be effective in delaying the spread of bacterial resistance.

## Introduction

Bacterial resistance to antibiotics has been growing and poses an increasingly serious threat to modern medicine [1], [2], [3]. Reducing unnecessary antibiotic use can lower the burden of resistant pathogens, but it is difficult to balance this against the benefits of antibiotics, real and perceived. Recent studies have shown that strategies such as multiple first line treatments or drug combinations can be effective in delaying resistance [4], [5], [6]. Optimized dosing and duration of antibiotic regimens could play a similar role in minimizing resistance without the need to deny treatment to patients for whom therapy helps insure against low-probability, high-consequence outcomes, such as mastoiditis in children with acute otitis media. However, these approaches have received relatively less attention. The relationships between drug dosing, treatment duration, drug pharmacokinetics and pharmacodynamics, and therapeutic efficacy that would inform decision-making are only now beginning to be understood [7]. The benefits to the individual, however, may be better evaluated by also considering broader immunological, epidemiological and ecological consequences of antibiotic use.

The optimal duration and dosing of antibiotic treatment is influenced by the dynamics of infection and immunity. A drug regimen must be given at a sufficiently high dose and sufficiently long duration to clear an infection. Since dosing must be low enough to avoid toxicity, the recommended drug dose is typically set just below the maximally tolerated dose and, consequently, in some cases, just slightly above the minimum concentration required to clear infection [8]. However, as resistance increases, so do the minimum inhibitory concentrations (MICs) and the amount of drug required to achieve a cure. It has been suggested that drug dosing that yields concentrations above the minimum required for inhibitory effect of the most resistant bacteria throughout the treatment would result in less selection for resistance [7]. Immunity is a strong ally in this process, since a strong immune response can significantly limit the need for long-duration treatments, and indeed, there has been a tendency towards shorter treatment regimen for some common acute infections [9], [10], [11], [12], [13], [14], [15], [16] in patients with intact primary and secondary host defenses.

The propensity of pathogens to also colonize other parts of the host without causing disease should also be relevant to the design of the appropriate treatment profile. Most bacterial pathogens are able to colonize the upper respiratory tract, the skin, the gut, or other tissues without causing disease. When bacteria infect sterile tissues, transmission is generally inefficient. For example, the methicillin-resistant *Staphylococcus aureus* isolates infecting deep tissue spaces and that cause septicemia are not shed and are therefore not infectious, while the staphylococci colonizing the skin of the same host are well positioned to so in a population through skin-to-skin contact. The public health perspective would place value in optimizing drug regimens for bacterial infections that lead to treatment success in the patient but doing so in a way that minimizes the risk of resistance in the ecological reservoir. Pharmacodynamic and pharmacokinetic properties of many antibiotics differ at the infection and colonization sites, making it more difficult to select one regime that achieves both objectives. Presently we lack the means to predict whether adjustments in the delivered dose, and the impact of that adjustment on successful therapy of the patient's infection, alter the risk to the public health related to the development of resistance in the colonizing and potentially transmissible flora.

Here we evaluate optimal dosing by considering a broader picture of the factors that influence the selection of antibiotic resistance. Important differences among bacteria suggest that ecological theory and mathematical models can help identify and frame the relevant issues. To this end, we have explored simple pharmacokinetic and pharmacodynamic (PK/PD) within-host models to determine optimal antibiotic dosing strategies that simultaneously minimize morbidity and selection for resistance. The models are used to compare the outcome of treatment regimes and classify pharmacodynamics across a broad range of conditions that, unlike earlier studies, consider how the drug might act differently on infecting and colonizing bacteria. These simulations were used to provide a theoretical basis for shorter dosing regimens, identify important parameters that disproportionately influence the optimal strategy, and establish the basis for a broader agenda for designing drug treatment strategies to slow resistance.

## Methods

To describe a broad range of possible dynamics of host and pathogen that may be clinically relevant, and to understand the way those interactions are altered by the presence of a drug given at varying effective concentrations, we use a model of bacterial population dynamics based on one described by Austin and Anderson [17]. The model considers competition between drug-sensitive and drug-resistant bacteria with population sizes of *S* and *R*, respectively. These populations are limited to some extent by an immune response *I*. The model assumes that bacteria grow at the rate λ, but a fraction, μ, of the sensitive bacteria become resistant through mutations. Sensitive and resistant bacteria die at the rates ξ_S_ and ξ_R_, respectively, and ξ_R_>ξ_S_ because of a biological cost of resistance. Growth of both bacterial populations is limited by the maximum population size *K*, and population sizes closer to the maximum population size imply reduced growth rates. The functions f_R_ and f_S_ describe the relation between antibiotic concentration and antibiotic effect on bacteria–i.e., the pharmacodynamics. The immune response grows in response to the bacteria challenge: *a* is the maximum per capita proliferation rate, *b* is the bacterial population that gives half the maximum rate, and *1*/δ is the average duration of the immune response. Killing of bacteria by the immune response is assumed to occur in the same manner regardless of resistance at a rate directly proportional to the strength of the immune response with a killing rate constant γ.

The dynamics are described by the following coupled ordinary differential equations:
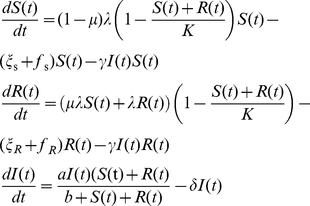
(1)


### Antibiotic Therapy, Resistance and Clinical Outcomes

In order to consider the best case for dosing to clear infection while minimizing resistance, we assume there are no natural constraints on pharmacokinetics and that drug concentrations are maintained at a constant level throughout treatment. Given a concentration *c*, the antibiotic effect on bacteria is described using an *E_max_* model [18], [19], [20], [21], [22], [23],
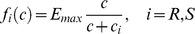
(2)where E_max_ is the maximum effect and *c_i_* is the drug required to produce half of the maximum effect for strain *i*. With *c_R_>c_S_*, resistant bacteria are not fully resistant to the drug but instead require a higher drug concentration for clearance.

The effect of drug pressure on selection for resistance varies by dosage and treatment duration, but also depends on the dynamics of target and non-target bacterial flora. Each bacterial specie can have different dynamics in each of the habitats it inhabits. Therefore, the tissue concentration of antibiotics reached at the infection site may not be equal to the concentration reached at the colonization sites. We varied drug levels and their effects to simulate selection on bacteria under a range of conditions that could be encountered by bacteria under antibiotic drug exposure. Concentrations were varied from below the MIC of the susceptible strain to above the MIC of the resistant strain. With μ = 0 and the interpretation that the MIC corresponds to the concentration that just inhibits bacterial growth, the MIC can be expressed by 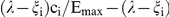. Parameters for the simulations were chosen to achieve a weak and strong drug action, defined by 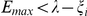 and 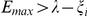, respectively.

Although the terms “colonization” and “infection” are clinically meaningful, they do not capture aspects of disease dynamics important for understanding resistance. We have therefore used four arbitrary but hopefully helpful definitions of infection dynamics that are motivated by a formal stability analysis of the mathematical model (see Text S1 for more details): unregulated, regulated, opportunistic, and self-limiting bacterial dynamics.

#### Unregulated dynamics

The interaction between the bacterial population and the host is insufficient to stimulate an immune response and the resistant and sensitive bacteria settle to equilibrium. This type of interaction could occur among bacteria that use the body primarily as a commensal habitat [24]—e.g. commensal staphylococcal flora of the skin. While those organisms may interact with the specific cell surface of epithelial cells, without an interruption of the epidermal layer, the organisms in this state do not provoke an immune response. Other examples of this type of dynamics could include a variety of potential pathogenic organisms that colonize the gastrointestinal tract, such as Helicobacter species, prior to causing invasive disease.

#### Regulated dynamics

Bacterial growth is followed by an immune response and both the bacterial populations and the immune response oscillate before settling to equilibrium. Continuing on the example of staphylococci, should there be an interruption of the epidermal layer, staphylococci numbers will increase and an immune response will be generated. To the degree that the epidermal layer is repaired, the organism burden will drop back to the original levels after a burst of replication. To the degree that the epidermal layer is not repaired, organism counts may remain higher than baseline, ultimately achieving a new equilibrium state. Clinical conditions ranging from trauma, to underlying fungal infections to atopic dermatitis and eczema [25] could all interrupt the intact skin layer and trigger this type of dynamic. Another clinical example could include infection with *Mycobacteria tuberculosis*, where the initial pulmonary exposure to the organism results in mild, localized disease which is checked by the immune system but results in an immunologic détente, in which the relatively dormant organism is now encased within a granuloma, precluding continued replication but failing to fully eradicate the threat.

#### Opportunistic dynamics

Bacteria arrive at a stable regulated equilibrium under drug exposure, but as the initial immune response develops, bacteria are driven down to very low densities that are below a cutoff value of one organism defined for eradication (see definition below) without drug exposure. Again, in the case of a skin infection, drug exposure has reduced the burden of organisms, as well as the cellular and immunotoxins generated by the staphylococci, in the locally infected site, allowing the immune response to return and maintain the bacterial count at levels even below the original colonizing state. Within the respiratory tract a similar dynamic may occur between Streptococci pneumoniae and the local mucosal host defenses. Although *S. pneumoniae* can reside within the respiratory tract without causing disease, should symptomatic infection occur as a consequence of an increasing burden of organisms, antibiotic intervention may drive down the number of organisms to levels at or below that consistent with eradication.

#### Self-limiting dynamics

Bacteria are driven down by the immune response to an equilibrium density below the cutoff. In a sense this is a specific outcome among the regulated dynamics in which the organism burden is essentially eliminated. For example, the invading staphylococci are destroyed by a combination of primary and secondary immune responses, without antibiotic intervention. In another clinical example, bacterial overgrowth and invasion triggers an immune response in the middle ear sufficient to reduce the bacterial burden to levels at or below the equilibrium density, with or without symptoms.

**Figure 1:**
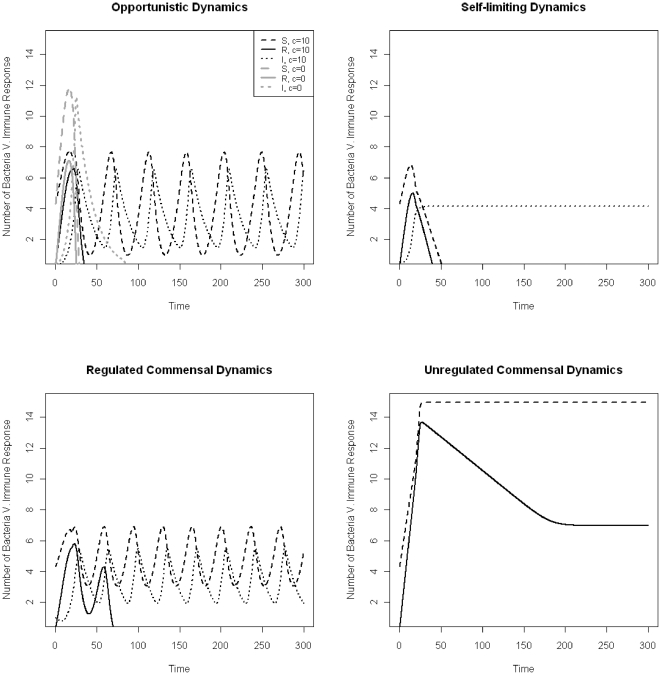
Simulation results showing the bacterial counts for the four different dynamics, assuming two different drug concentrations (c = 0 black, and c = 10 grey). Unregulated dynamics: No immune response is stimulated in the case of unregulated dynamics and the resistant and sensitive bacteria settle to equilibrium. Regulated dynamics: both bacterial populations and the immune response oscillate before settling to equilibrium. Opportunistic dynamics: bacteria end up at a stable regulated equilibrium under drug exposure, but are driven down to very low densities that are below the cutoff value defined for eradication without drug exposure. Self-limiting dynamics: bacteria are driven down to an equilibrium density that is below the cutoff value by the immune response.

Opportunistic and self-limiting dynamics differ in that, in the case of opportunistic dynamics, re-exposure to bacteria in the presence of a pre-existing, primed immune response could lead to a stable regulated dynamic but with an equilibrium value above the cutoff defined as ‘eradication’, while the self-limiting infection will always result in the eradication of the organism. Population densities of regulated or unregulated commensal dynamics tend to be close to their population dynamic equilibrium at the time of treatment, while opportunistic and self-limiting dynamics are typically in the geometric growth phase of the infection when treatment starts. The differences between these types of pathogen dynamics and their simulations are illustrated in Figure 1.

For each of these types of bacterial dynamics, we analyze three different outcomes; aggregated resistant bacterial load (defined as the integral of R(t)), the aggregated fraction of resistance (defined as the integral of R(t)/(R(t)+S(t))) and the time with symptoms (defined as time with bacterial loads >10^7^).

### Cutoff Value

To obtain more realistic results from our simulations, we have defined a cutoff value of one bacterium as the minimum value required for survival of a bacterial population. Once the last bacterium has died, the population has been eradicated.

## Results

**Figure 2:**
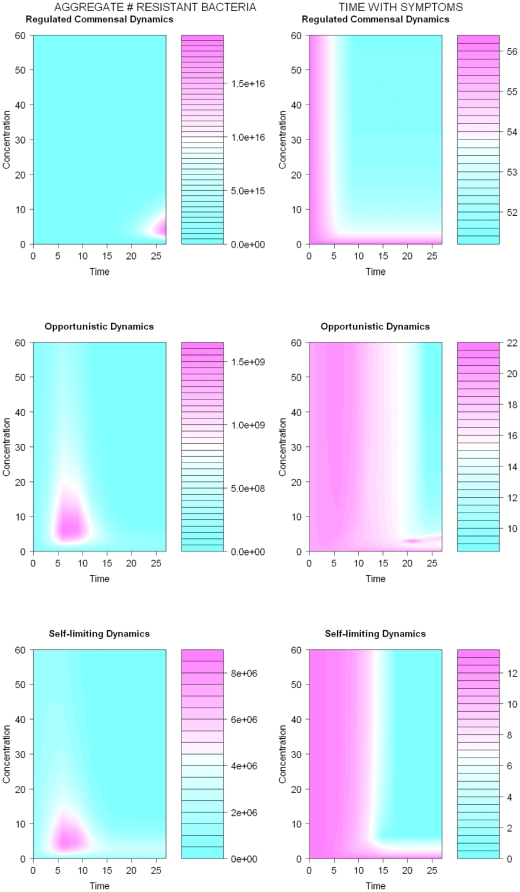
The outcomes of different dosing strategies on the aggregate number of resistant bacteria (left panel) and time with symptoms (bacterial loads >10^7^, right panel) are contrasted for regulated, self-limiting, and opportunistic bacterial populations. The antibiotic concentration is assumed to be constant throughout the treatment at the concentration displayed by the y-axis for a time displayed by the x-axis. The pink color indicates the area with the strongest selection for resistance and the longest durations with symptoms. Resistance increases with longer durations of antibiotics for regulated commensal. For opportunistic and self-limiting dynamics, the selection for resistance is most intense for intermediate durations of antibiotics. The time with symptoms decreases with increased durations and concentrations of antibiotics for all types of bacterial dynamics. The time with symptoms is relatively short for opportunistic and self-limiting dynamics compared to that of regulated commensal dynamics. This illustrates the importance of the immune system for the clearance of bacterial populations. Simulation results for unregulated dynamics (not displayed) are similar to that of regulated dynamics but selection for resistance is somewhat stronger and time with symptoms somewhat longer. Parameters used to achieve regulated commensal dynamics are *E_max_* = 1, *a* = 0.2, *K* = 10^15^, λ = 1.8, *c_50R_* = 13.1, *c_50S_* = 1.3, γ = 50, *b* = 10^6^, δ = 50, μ = 10^−8^, ξ_R_ = 0.2, and ξ_S_ = 0.1. For unregulated commensal dynamics and opportunistic infection dynamics, δ = 0.05, and for self-limiting infection dynamics, δ = 5 ⋅ 10^−8^. A small subpopulation of bacteria is resistant at start. The initial value for the immune response is zero for the unregulated commensal dynamics and is close to the equilibrium (≠0) for other cases. Antibiotic concentration is kept fixed at the concentration displayed by the y-axis for a time displayed by the x-axis. All parameters are given in arbitrary units.

Selection for antibiotic resistance and clinical outcomes differ, depending on the ecological dynamics of the pathogen in relation to dosing and duration of treatment (Fig. 2).

### Optimizing treatment for regulated or unregulated pathogen dynamics

If pathogens, whether regulated or unregulated by the immune response give rise to symptoms, antibiotics must be relied on for elimination of symptoms, and high drug doses for long durations will be required (right panel, Fig. 2). Clinical examples include tuberculosis, acne and *Helicobacter pylori*. These chronic or recurrent infections differ in terms of severity of symptoms but require prolonged or repeated antibiotic therapy/prophylaxis. If resistant subpopulations are initially present (as is the case in our simulations) or arise through mutation or genetic transfers during the course of treatment, the frequency of resistance will correlate with the time that a drug concentration is maintained between the MICs of the susceptible and resistant strains (left panel, Fig. 2), consistent with findings from *in vitro* pharmaco-kinetic studies [26], [27]. Although mutants with MICs between the sensitive and resistant strains play a major role in the evolution of resistance, we also show that the frequency of resistance increases with duration of antibiotic treatment for the other dosing strategies. We arrive at similar qualitative conclusions regardless of whether the outcome measures are in terms of aggregated number or aggregated fraction of bacteria (figures not shown).

While resistance evolution of regulated or unregulated infections is a significant issue for some infections, the impact of antibiotic treatment on commensal bacteria is often overlooked. Resistance in commensals is clinically important, because each episode of treatment places selection pressure in favor of resistance in both the infection and the commensal sites. Moreover, for a given level of drug concentration, selection is more extensive in commensals because of the lack of a strongly limiting immune response. Regardless of pathogen dynamics, it is unlikely that antibiotic treatment can successfully clear colonization of the host due to the large numbers of organisms in the commensal flora. Antibiotic resistant bacteria are more likely enriched in the commensal flora of patients with high antibiotic usage [28].

Although large, stable commensal bacterial populations are driven toward fixation of resistance under antibiotic pressure, the frequency of resistant organisms declines to a mutation-selection balance when the drug pressure is relaxed, by assumption, as a result of the fitness cost related to genetic reassortment (see Text S1). This balance may be further complicated by the presence of factors not considered here, such as compensatory mutations, which reduce the biological cost of resistance [29].

### Optimizing treatment for self-limiting or opportunistic pathogen dynamics

Self-limiting pathogen dynamics differ from regulated or unregulated dynamics in several aspects. First, as the name suggests, self-limiting infections are inherently time-constrained and the time with symptoms is substantially shorter relative to deep tissue infections, with or without antibiotics. Important clinical examples include some of the most common infections, acute otitis media (AOM) and acute sinusitis, for which empirical studies have shown that no treatment or a short treatment of three days is efficient for curing the infection [11], [12], [15], [16], [30], [31], [32]. As is seen in Figure 1 (compare opportunistic dynamics with and without drug), drugs can change the dynamic outcomes. Opportunistic infections may resemble self-limiting infections in their dynamics without drug exposure, but drugs can change the dynamics to end up at a stable regulated equilibrium. The resulting chronic colonization or infection could have long-term consequences on the patient's health. These dynamics could possibly explain the empirical findings that recurrent otitis media occurs more often in children treated with antibiotics than those untreated [33].

**Figure 3:**
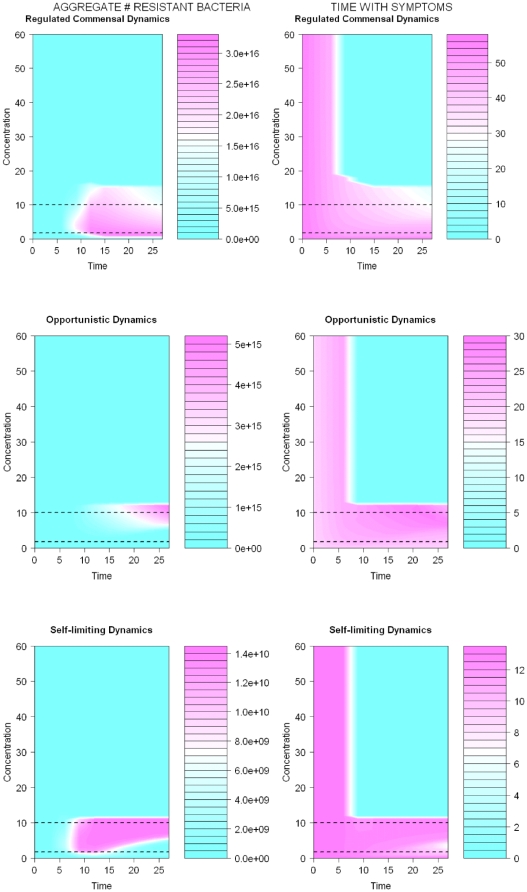
The outcomes of different dosing strategies on the aggregate number of resistant bacteria (left panel) and time with symptoms (bacterial loads >10^7^, right panel) are contrasted for regulated, self-limiting, and opportunistic bacterial populations. The antibiotic concentration is assumed to be constant throughout the treatment at the concentration displayed by the y-axis for a time displayed by the x-axis. The pink color indicates the area with the strongest selection for resistance and the longest durations with symptoms. Resistance increases with longer durations of antibiotics for regulated commensal dynamics. For opportunistic and self-limiting dynamics, the selection for resistance is most intense for intermediate durations of antibiotics. The time with symptoms decreases with increased durations and concentrations of antibiotics for all four types of bacterial dynamics. The time with symptoms is relatively short for opportunistic and self-limiting dynamics compared to that of regulated commensal dynamics. This illustrates the importance of the immune system for the clearance of bacterial populations. Simulation results for unregulated dynamics (not displayed) are similar to that of regulated dynamics but selection for resistance is somewhat stronger and time with symptoms somewhat longer. The E_max_ parameter was set to 3 to contrast the results for a situation with strong action by drugs. The other parameters used to achieve regulated commensal dynamics are a = 0.2, K = 10^15^, λ = 1.8, c_50R_ = 13.1, c_50S_ = 1.3, γ = 50, b = 10^6^, δ = 50, μ = 10^−8^, ξ_R_ = 0.2, and ξ_S_ = 0.1. For unregulated commensal dynamics and opportunistic infection dynamics, δ = 0.05, and for self-limiting infection dynamics, δ = 5 ⋅10^−8^. A small subpopulation of bacteria is resistant at start. The initial value for the immune response is zero for the unregulated commensal dynamics and has a value close to the equilibrium (≠0) for the other cases. All parameters are given in arbitrary units. Lower and upper dashed lines represent the MICs of sensitive and resistant bacteria, respectively.

Second, for both self-limiting and opportunistic infections the selection for resistance (in aggregate numbers of resistant bacteria) is most extensive for intermediate dosing strategies near the MIC for intermediate durations (see Fig. 2). The results presented assume a weak action by the drug. The dynamic differ slightly when the action by the drug is strong (see Fig. 3), but our conclusions are robust to these variations.

Thus, for infections caused by organisms which are commensal in their normal habitat, the best strategy would be a short and aggressive treatment early in an infection to keep bacteria populations low until the immune system can finish the job—because this strategy will minimize the selection for resistance among the commensals at the same time as minimizing selection in the infection site. The tradeoff of choosing the strategy that minimizes resistance is that the time with symptoms becomes longer with shorter durations of antibiotics.

## Discussion

Recent developments in the field of antibiotic pharmacodynamics have led to a better knowledge of the optimal dosage strategies to obtain maximal eradication of the infecting pathogen [34], [35]. Further, optimizing the duration and dosing of treatment to reduce the likelihood of resistance for a given level of antibiotic use, however, remains a challenge. Resistance to dozens of antibiotics has evolved in hundreds of bacterial species, but the relationships between dosage regimens, pharmacokinetics and therapeutic efficacy in the context of bacterial resistance are only now beginning to be understood [7]. The public health response to various aspects of the resistance problem has largely focused on a single solution—the reduction of antibiotic overuse. A more holistic approach may be to build up a functional taxonomy, a classification of bacterial pathogens that are functionally similar for resistance evolution. A functional taxonomy would consider pathogen ecology, the dynamics of immunity along with other issues that are important determinants of transmission and of population structure. Those other important concerns may include the typical population sizes of colonizing bacteria, their intrinsic resistance to antibiotics, their tendency to accumulate resistance genes, and species-specific cross-resistance to dominant antibiotics.

Our research particularly emphasizes the public health importance of understanding the dynamics of bacterial pathogens in their dominant ecological reservoir, and pharmacodynamic and pharmacokinetic parameters operating in those tissues, as an important complement to concerns about optimizing treatment for bacterial infections.

In this paper, we show that 1) all antibiotic use—even under a “perfect” dosing strategy—exerts selection for resistance, and 2) for most infections the optimal dosing strategy for clinical treatment may not be optimal for preventing the spread of resistance, likely a consequence of those treatment regimens may not being focused on the dynamics of bacterial populations. The general rule suggested by our studies is that shorter duration of treatments is usually, but not always, optimal. More comprehensive guidelines that consider the important differences among bacteria in their ecological dynamics, as well as in the diversity of ecological dynamics for the same bacteria in different habitats in the body could contribute to dramatically reduce the volume of antibiotics consumed and selection for resistance.

An example is the treatment of AOM—the second most common infection among children after the common cold, and the single most important reason for antibiotic prescriptions in the United States [36]. Here, no treatment or a short three-day course with antibiotic treatment has been shown to be no less effective than ten days with antibiotics [12], [30], [31]. The motivation for the trend and steady progress in designing shorter drug regimens have been based on clinicians' experience rather than on systematic evaluation of the trade-off between treatment success and resistance, and yet, many physicians continue to recommend that patients complete the full 10 day course of antibiotic treatment [37], [38], thereby potentially accelerating the rate with which resistance evolves and spreads from other bacterial populations. A guideline change toward shorter treatment durations with antibiotics would not only prevent resistance among individuals receiving antibiotics for self-limiting infections, but also among the large number of patients receiving antibiotics for nonbacterial infections [39].

For infections that cannot be handled by the immune system alone, i.e. for which high concentrations of antibiotics for long durations are required, it is important to consider the functional taxonomy of the drugs used. The mode of administering the antibiotic is important because it affects the concentrations of the drug in other parts of the body; antibiotics typically reach much higher concentrations in the gut if given orally rather than intravenously [40]. For instance, there is evidence suggesting that the rise of vancomycin-resistant enterococci in the United States during the 1980s could have been driven by the use of oral vancomycin for *C. difficile* [41]. In Europe, where vancomycin was mostly used intravenously, vancomycin concentrations and selection for resistance in gut commensals would be expected to be much lower, and vancomycin-resistant enterococci are much less frequent. Tuberculosis treatment does not impose an increased selection for resistance, despite extensive drug regimes, because *Mycobacterium tuberculosis* does not form a part of the commensal flora, and because the two key agents (izoniazid and ethambutol) in the triple-drug combination are active only against mycobacteria, which are not a part of the commensal flora. In contrast, for the treatment of acne with broad-spectrum antibiotics (minocycline), sometimes for a year or more [42], our results suggest a significant selection pressure on the commensal flora. By leaving the gut flora, upper respiratory flora, and much of the skin flora unexposed, topical usage of antibiotics could be a solution. It has, however, long been discouraged and amounts to only 1% of systemic use [39].

Since populations of commensal flora can be extensive, resistant bacteria generated by otherwise rare mutations or genetic exchange events are likely to exist and comprise some part of the existing flora at the time of antibiotic treatment [43]. Resistant commensal bacteria are therefore more likely to persist and cause resistant infections when they spread to other hosts. Even if they do not cause infection, commensal bacteria can transfer genetic material coding for resistance to other, more pathogenic bacteria.

It follows that the value of drug optimization is related to the full spectrum of drug pharmacokinetics and pharmacodynamics throughout the body, both in terms of the delivery mode and the organisms it targets. Optimization of treatment in the primary ecological reservoir for transmission is both the best measure of collateral damage in transmission models and the most important consideration for public health. Our results emphasize the need to reexamine topical, intravenous, or focal delivery of antibiotics for other drug and organism combinations.

### Caveats and Limitations

Like all mathematical models of biological systems, the models analyzed here involve some assumptions and simplifications. Mutations in the model are generated through a one-step process. Genetic transfers typically result in higher levels of resistance, but are unlikely to occur in self-limiting infections unless there is a coinfection; the first documented case of infection caused by vancomycin-resistant *S. aureus* is one important example [39]. Allowing for mutations to high-level resistance at the colonization site increases the time span in which there is extensive selection for resistance, but does not alter our conclusions.

We assumed drug concentration were maintained at a constant level throughout treatment—an assumption which is unnatural for most situations other than for intravenous treatment [18]. For oral and intramuscular administration, the concentrations of antibiotics will be in a continuous state of flux, and the dynamics could be further confounded by factors such as non-compliance. As result, the effect of periodic waning of antibiotic concentrations below the effective MICs are not considered in our analysis, but instead constant concentrations either below or above the effective MICs are considered. Accounting for periodic waning of antibiotic concentrations to below the effective MICs will affect the intensity of the selection for resistance and the time scale for the dynamics and should therefore be considered in future analyses for antibiotic and pathogen specific guidelines.

The MIC was used as a single pharmacodynamic parameter in the model. The pharmacodynamic function captures the effect of an antibiotic over a wide range of antibiotic concentrations and as a consequence of differences in the shape of these pharmacodynamic functions, antibiotics with the same MICs and pharmacokinetics may differ profoundly in their microbiological efficacy [23]. This assumption may have to be relaxed for future predictions in the development of antibiotic treatment protocols for specific drug and organism combinations.

An important aspect of our study was that we explicitly considered the different dynamics of drugs and bacteria that could occur at the infection site and in other habitats, taking into account the effects of immune response on the emergence of and selection for resistance. Bacteria populations in different habitats in a body are not expected to have strong direct interactions, so the population responses would be different and largely independent. Because of the many interdependent mechanisms, such as different cell types and cytokines involved in the immune responses to bacterial infections, experiments to measure the contribution of various components have been difficult, and precise knowledge about these processes is still lacking [44]. The focus of this study, however, was not prediction of resistance emergence for a specific infection or species of the microflora, but rather a conceptual framework for addressing questions about the impact of considering both the infection site and the commensal site in the optimization of antibiotic drug dosing regimens to prevent resistance.

In the model, the immune response depends on the total bacterial population. There is, however, evidence that some pathogens may escape the effects of an antibiotic by moving from the extracellular to the intracellular space. In contrast to our assumption, resistance to an antibiotic would then be correlated with increased resistance to the immune response [45], [46]. The evidence for this phenomena is still emerging and further investigation is needed. Were this observation to become established, however, the degree of resistance prior to treatment could affect the overall immune response.

There may be other important clinical outcomes to consider in the evaluation of dosing strategies. Some studies for instance have reported an increase in the number of complications from countries with lower rates of antibiotic prescribing for AOM [47]. The incidence is low, however, and the risk of more serious sequelae has to be weighed against the risk and consequences of a strategy that generates more-resistant organisms.

Lastly, this study only captured one of the two overlapping problems related to resistance: the emergence and selection of resistant strains within the host. For many infections, primary resistance caused by the spread of resistant strains within a population is the most significant problem [48] and it remains important for future studies to evaluate whether the gain of treating a patient outweighs the risk of resistance in the population.

### Summary and Conclusions

Existing antibiotic treatment guidelines do not consider 1) important differences in the ecological dynamics among different bacterial species or 2) the diversity of ecological dynamics within the same bacterial species in different habitats in the body. Our results challenge the conventional clinical wisdom that long durations of antibiotic therapy are appropriate for common infections regardless of whether or not they are self-limiting, and regardless of the potential for selection for resistance in the commensal flora. In conclusion, one-size-fits-all dosing regimens do not fit all combinations of organisms and antibiotics, demanding a need to broaden the principles employed in selection of dosing regimens for both existing and future antibiotics to include not only successful treatment of the underlying infection but doing so in a manner that minimizes the pressure to select for increasingly more antibiotic resistant pathogens.

## Supporting Information

Text S1 **Equilibrium points and stability analysis.**
(DOC)
