# Supplementary material for: “One-Size-Fits-All”? Optimizing Treatment Duration for Bacterial Infections
Source: PLoS One. 2012 Jan 11;7(1):e29838. doi: 10.1371/journal.pone.0029838 (PMC3256207; doi:10.1371/journal.pone.0029838)
Supplement: Text S1 — Equilibrium points and stability analysis. (DOC) [file pone.0029838.s001.doc]

**Supporting information**

**Equilibrium points and stability analysis**

Because of the limited duration of drug dosing, the equations are most often solved numerically to evaluate the time course of a single infection under treatment. In the sections below, we have conducted a formal stability analysis, where we regard the steady states as attractors that show where the system is tending, if the current conditions remained stable, and that highlight the different ecological dynamics at work.

The analysis of Equations (1) is simplified by introducing the variable change, *T = S+R* and *x = S/T*. For notational simplicity, we also let A = ξ_S_ + f_S_ and B = ξ_R_ + f_R_. Equations (1) can then be rewritten in the new variables as:


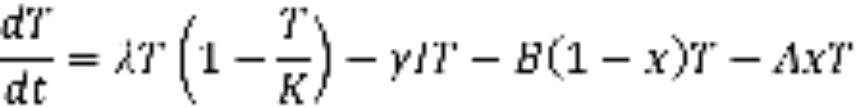


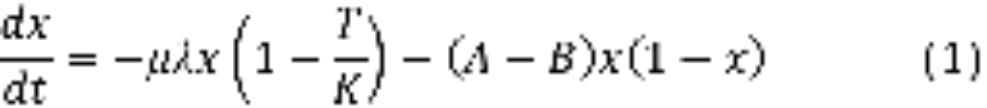


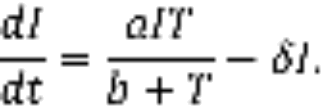


If μ = 0, the evolutionary dynamics lead to fixation or extinction of resistance, depending on whether *A>B* or *B<A*. The terms *A* and *B* describe the net death rates of each type. In general, because of the biological cost of resistance, we assume that *B>A* under antibiotic drug pressure and that *B < A* without drug pressure. In this case, the balance of selection is also affected by the mutation rate for resistance, μ, that tilts the fitness advantage towards resistance and that guarantees resistance is present at some low frequency. In ecological dynamics that lack an immune response, bacterial competition for resources limits population densities. Resistance fixes if *B(1-μ) < A*. In the presence of an immune response, the condition for resistance to fix is B(1-μ) *< A* + μγ*I*; resistance fixes for a slightly broader range of parameters (not necessarily more easily) when the bacterial populations are regulated by immunity instead of competition.

The different ecological and evolutionary dynamics that may arise, depending on the parameters, are summarized by the following equilibrium points and stability analysis:

1. If λ (1-μ) < *A* and λ < *B*, then bacterial death rates exceed the growth rates and no bacterial populations can be sustained. The equilibrium, denoted Q^(0)^, is described by the triplet *(T,x,I)* = (0, 0, 0), and it is stable. This situation reflects a body habitat which is unfavorable for bacterial growth.
2. If there are no drug-sensitive bacteria present initially, or in other words, if resistance is fixed by the initial conditions, and if λ > *B*, then some resistant bacteria will be present. Resistance will remain fixed, because the equations ignore the possibility of back mutations to a drug sensitive type, and the equilibria and their stability follow the analysis described above. Fixation of resistance may or may not be stable to perturbation, such as the introduction of sensitive bacteria from an exogenous source, depending on conditions described in the analysis below.
3. If λ (1-μ) > *A* or λ > *B*, then the expression for the equilibrium depends on whether the bacteria population stimulates an immune response, which depends, in turn, on the equilibrium bacterial densities and the kinetics of the interaction with the immune system. These equilibrium densities stimulate an immune response if the equilibrium densities, denoted
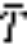
, exceed the threshold δ b / (*a* - δ). Expressions for
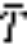
 follow:
   1. If *B*(1-μ) < *A*, then resistance fixes (i.e.,
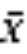
 = 0). Bacterial population densities reach the equilibrium density
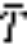
 = *K* (*1- B* / λ). This equilibrium, denoted Q^(1)^ = (
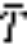
, 0, 0), is stable and Q^(0)^ exists but is unstable.
   2. If *B*(1-μ) > A, then the frequency of resistance reaches a stable equlibrium 1-
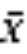
 =
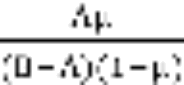
. Bacterial population densities reach the equilibirum
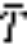
 = *K* (1- [ *B* (1-*x*) - *A x*] / λ). This equilibrium, denoted Q^(2)^ = (
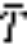
,
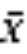
, 0) is stable, and Q^(0)^ and Q^(1)^ exist but are unstable.
4. If λ (1-μ) > *A*, and if (*a*-δ)
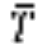
 > δ *b*, then the bacterial populations reach an equilibrium population density that is sufficient to generate an immune response. The bacterial populations are then regulated by the immune response at the level
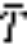
 = δ *b /* (*a*- δ).
   1. If (*B-A*) < μ λ ( 1-
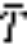
/*K*), then resistance will fix and the equilibrium immune response is
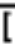
 = ((1-μ) λ ( 1-
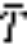
/*K*) -*B*) / γ. This equilibrium, denoted Q^(3)^ = (
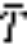
, 0,
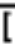
) is stable. Two other equilibria, Q ^(0)^ and Q ^(1)^, exist but both are unstable.
   2. If (*B-A*) > μ λ ( 1-
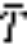
/*K*), then resistance does not fix, but reaches the equilibrium frequency 1-
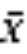
 =
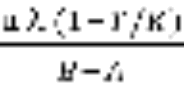
. The equilibrium immune response is
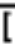
 = ((1-μ) λ( 1-
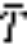
/*K*) –A) / γ. This equilibrium, denoted Q^(4)^ = (
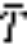
,
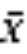
,
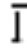
) is stable. Four other equilibria, Q^(0)^, Q^(1)^, Q^(2)^, and Q^(3)^, exist but are unstable.
